# The Potential Use of Plant Growth Regulators for Modification of the Industrially Valuable Volatile Compounds Synthesis in *Hylocreus undatus* Stems

**DOI:** 10.3390/molecules28093843

**Published:** 2023-05-01

**Authors:** Maciej Jakobina, Jacek Łyczko, Kinga Zydorowicz, Renata Galek, Antoni Szumny

**Affiliations:** 1 Department of Plant Breeding and Seed Production, University of Environmental and Life Sciences, Grunwaldzki Square 24a, 50-363 Wrocław, Poland; 116404@student.upwr.edu.pl (M.J.);; 2 Department of Food Chemistry and Biocatalysis, Wrocław University of Environmental and Life Sciences, Norwida 25, 53-375 Wrocław, Poland

**Keywords:** pitaya, chemical compounds, in vitro cultures, modification synthesis, phytohormones

## Abstract

The pitaya (dragon fruit) *Hylocereus* is a genus which belongs to the Cactaceae family. It is native to Mexico, occurring also in other regions of Central and South America. Pitaya fruit is mainly intended for consumption and for this reason the species is grown commercially. The fruit is a rich source of vitamins, biologically active compounds, and dietary fibre. Using in vitro culture can accelerate the process of reproduction and growth of pitaya plants. Profiling of volatile compounds contained in the stem of *Hylocereus undatus* was carried out using the SPME-GC-MS technique. The main compounds present were hexanal, 2-hexenal and 1-hexanol. The results showed differences in the occurrence of volatile compounds between plants grown in media with an addition of BA (6-benzylaminopurine) and IAA (indole-3-acetic acid), which have been used as plant growth regulators. Statistically significant differences between the contents of volatile compounds were observed in the case of 2-hexenal and 1-hexanol. The effect of BA on reducing the amount of volatile compounds was observed. However, introduction of IAA to the in vitro medium resulted in more compounds being synthesized. This study is the first to describe the volatile compounds in the pitaya stem. The results indicate that plant hormones are able to modify the profile of volatile compounds.

## 1. Introduction

*Hylocereus* ssp. are perennial succulents, growing as epiphytes. The genus originates from tropical and subtropical forests of Mexico and other areas within Central and South America (e.g., Guatemala, Salvador, Panama, Costarica, Colombia or Brazil). From its native localities the plant has been spread to Near East and Australia. Nowadays, the industrial plantations of pitaya are mostly located in Asian countries, namely, Thailand, Malesia and Taiwan. The pitaya is a cactus genus characterized by long triangular green stems, equipped with thorns on the edges. The fruit is an elongated berry. The peel’s characteristic feature is intense dark pink or reddish purple coloration with green scales resembling leaves. Pitaya fruit is mostly used for consumption and therefore the plant is cultivated for commercial purposes. It is a rich source both of vitamins (C—20.5–33 mg/100 g pulp, B3—0.2–2.8 mg/100 g pulp) and dietary fibre (69 g/100 g of dried pulp). Its pH value reaches 4.4–5.1. The predominating acid is malic acid [1,2].

Full production of raw material falls on the fifth year of land use. It is possible to incessantly obtain fruit for 10–15 years. Depending on the region of cultivation, the yield from 1 ha plantation can reach as much as 16–80 tons of fruit annually [2,3,4,5,6,7,8,9]. Plantlets for establishing a plantation can be acquired on generative way from seeds and vegetatively from in vivo or in vitro cultures. The seedlings raised from seeds grow slower and need a longer time to reach the reproductive phase, while the individual specimens obtained are diverse. The second method, which is easier and cheaper, is vegetative propagation by cuttings. It consists in in vivo cutting of plants to acquire 15–60 cm-long segments (the longer the fragment the faster the regeneration time). Whereas using in vitro culture, one can accelerate the process of vegetative propagation and growth of the dragon fruit plants. Thanks to this method it is possible to acquire healthy pathogen-free plant material in a very short time [3,5,7]. So far research with the use of plant in vitro cultures in this species concentrated on acquisition of effective micropropagation and acclimatization [9].

From the perspective of different branches of bio-economy, modification of metabolic paths in plants finds a wide array of applications. Alterations to the chemical composition can be induced under in vivo and in vitro conditions. Increased production by plants of biologically active compounds can be applicable in industry. In the tissues of many *Hylocereus* species it was possible to enhance the production of betacyans through triggering auto-polyploidization and also by using tyrosine or methyl jasmonate, whereas the synthesis of betalins was intensified with the use of elicitors, tyrosine or leucine as well as when raising the plants in red light conditions Table S1 [10,11,12,13,14,15,16,17,18,19,20,21,22].

It was not only in species falling within the genus *Hylocereus* that synthesis of valuable chemical compounds was effectively enhanced. Table S1 provides examples of species representing different plant genera and families for which such modifications have been reported [23,24,25,26,27,28,29,30,31,32,33,34,35,36,37,38,39,40,41,42,43,44]. Besides, in species belonging to the genus pitaya, namely *H. monacanthus*, *H. undatus* and *Hylocereus polyrhizus*, as well as in represenatives of other plant genera, such as *Andrographis paniculata*, *Phoenix dactylifera* L. or *Artemisia argyi*, intensified production of flavonoids was successfully initiated Table S1. Consequences of the presence of plant growth regulators (PGR) and their possible impact on changes in the synthesis of volatile compounds in pitaya stems have not been analyzed as yet. So far modification to the profile of volatile compounds with the use of plant hormones was successfully performed in a few plant species representing other genera. Application of jasmonic acid in the narrow-leaved lavender (*L. angustifolia* ‘Luoshen’) under in vitro conditions caused an increase in the content of volatile substances—monoterpenoids and sesquiterpenoids Table S1. A similar effect was obtained in the *Pyrenees oak*, in which application of the above mentioned compound resulted in enhanced emission of volatile compounds, although the overall chemical composition did not change. In the case of the orange *Citrus sinensis* L. ‘Osbeck’ alterations in the contents of volatile compounds were recorded, but also emission of E-β-ocimen and indole was found intensified. Taking into consideration the present state of knowledge, the herein discussed research was an attempt at defining the impact of two chosen plant growth regulators representing different groups—BA (cytokinin) and IAA (auxin)—on the profile and percentage of particular volatile compounds in pitaya stems coming from in vitro culture. These plant hormones allowed pitayas to be maintained in in vitro cultures. In addition, there are reports on these regulators that may have consequences of secondary metabolites in plants [45]. The hitherto reported data on volatile compounds in the pitaya are very scarce in the literature.

## 2. Results

The main compounds occurring in *Hylocereus undatus* tissues coming from both types of culture were: hexanal (6.87–4.53%), 2-hexenal (22.53–25.48%) and 1-hexanol (40.89–36.44%). The profile of volatile compounds in plants bred on media with an addition of plant hormones differed. The plants which were cultivated on a medium supplemented with IAA were characterized by occurrence of the following compounds: 1-heptanol, 1-octen-3-ol, octanal, o-cymene, ethanol, 2-(2-butoxyethoxy), 2-decenal, tetradecane and geranyl acetone, which were not detected in plants bred on media enriched with BA. Whereas in the tissues of pitaya cultured in vitro on a medium containing BA heptanoic acid, octanoic acid and hexyl ester were detected, which were not found in plant tissues grown on a medium with IAA added. A clearly negative impact of BA on the synthesis of volatile compounds was observed. Unlike, addition of IAA to the cultivation medium induced synthesis of a larger number of compounds. Statistically significant differences in the shares of volatile compounds were revealed Table 1. Mean values have been qualified into seven homogeneous groups based on Tukey’s test. The mean values of the following compounds: 1-hexanol coming from plants cultivated on a medium with an addition of BA, 1-hexanol from plants bred on a medium enriched with IAA, 2-hexenal coming from plants growing on a medium with IAA and 2-hexenal from plants grown on a medium containing BA have been qualified with distinct homogeneous groups (a–d). Most compounds identified—taking into consideration plant growth regulators added—did not exceed a mean share of over 5%.

## 3. Discussion

In their research based on in vivo material of *Hylocereus megalanthus* fruit, the team of Quijano-Célis et al., (2012) [46] identified the same compounds as those found in the present study, namely hexanal, 2-hexenal, 1-hexanol, 1-octen-3-ol, octanal, hexanoic acid, limonene, 1-octanol, nonanal, octanoic acid, decanal, 2-decenal, nonanoic acid, tetradecane, geranyl acetone, 1-dodecanol. However, our research revealed occurrence of 2-decenal, tetradecane and geranyl acetone only in plant tissues cultivated on a medium enriched with IAA and not that containing BA. Compounds such as linear alcohols and aldehydes (LAs, e.g., 1-hexanol, 2-hexanal) that occurs in plant are synthesized mainly from ω-3 and ω-6 carboxilic acids via hyper-peroxidation reaction (LOX). It was found, that they could play an important role in plant defense against herbivores and pathogens. Although the biosynthetic pathway of Las is not directly triggered with auxins or BA mode of action Escobar-Bravo found the relation between emission of 3-hexenyl acetate with changes in auxin concentration in maize. Also [47] (2013) proven that BA and IAA could directly effect on LOX activity, and in LAs emission as a response to stress. In grapes [48]. proven that cytokins effect on hexenals and hexenols biosynthesis, although it’s pathway is unclear. Due to their activity, the most interesting volatile compounds, applicable in industry, including pharmaceuticals production or plant protection, which were detected in the tissues of *Hylocereus undatus* were hexanal, 2-hexenal, 1-hexenol, *o*-cymene, limonene, eucalyptol, β-linalool and thymol. 

Recent years, more and more volatile compounds from plants have been identified as protective compounds against pests [49,50,51]. Hexanal induces early apoptosis of saprophytic fungus (*Aspergillus flavus*) conidia and have actively inhibits the growth of *Aspergillus* and *Penicillium* species. In addition, it is one of the attracting compounds for *Holotrichia parallela* [52,53,54,55]. Another component of the fresh green fragrance is 2-hexenal found in green plants. It is a typical defense compound of many insects, but it is also a component of the pheromone in several insects of the genus *Podisus* and in the species *C. lectularius* [56,57,58]. 1-hexanol was identified during the analysis of volatile compounds of fruits, among others, of species from the genus *Pyrus spp*. And *Prunus spp*. Moreover, this compound has an antagonistic effect on the sex pheromones of *Adelphocoris lineolatus*, while it attracts the insect *Lobesia botrana* [59,60,61,62]. Regarding the activities of hexanal and 2-hexenal, the abundance of those compounds in in vitro cultivations of pitaya supported with growth regulators may be a valuable feature for biotechnological production of agents dedicated for pest and fungi management.

Moreover, the interesting result was the observation that samples cultivated on the medium with IAA were able to synthesis other compounds, like *o*-cymene than cultures with BA. *o*-Cymene is one of the major the main components (65.2%) of the essential oils from *Bursera simaruba* (L.) Sarg., *Thymus vulgaris* ((56.2%) and *Nigella sativa* L. seeds (37.82%). The latter essential oil exhibits anti-oxidative properties, anti-microbial effect against clinically significant strains of bacteria and fungi whereas the thyme oil possesses potential activity against fungi causing the brown rot disease, what again brings the perspective of use of in vitro pitaya cultures industrial importance, however it has to be highlighted that for this purpose further optimalization of the process would be required due to low share of this compound in the present study Besides, *o*-cymene shows anti-viral effect against the virus of human influenza H1N1 [63,64,65,66,67].

In the case of limonene, eucalyptol, β-linalool and thymol the difference was observed between BA and IAA cultures, in favor for BA ones—the share of listed volatiles were higher. First of them -limonene is the most important volatile obtained from citrus peels essential oils, in which the content of limonene can reach as much as 97–98%. Limonene has been reported to control the development of *S. aureus*. What is more, in pre-clinical investigations this compound was revealed to have an inhibitory effect on the development of cancers, including the melanoma. Studies in vitro yield similar results. Limonene is characterized by anti-viral activity against the bird flu virus H5N1 type A and against COVID-19. At present it is possible to acquire limonene from citrus wastes [68,69,70,71,72,73]. Moreover, *C. reticulata* leaf essential oil from cultivars Cara mandarin, Kishu mandarin and Willow leaf mandarin—whose one of the main components is d-limonene—was reported to have a promising inhibitory impact on the tested aging enzymes [74]. Furthermore, eucalyptol is a component of the essential oil of a few eucalyptus species, i.e., *E. longicornis* (84.2%), *E. wandoo* (73.6%), *E. Lesouefii* (40.8%). The essential oils from eucalyptuses are reported as highly effective in controlling the adhesion by gram-negative (*Pseudomonas aeruginosa, Escherichia coli* and *Acinetobacter baumannii*) and gram-positive (*Staphylococcus aureus* and *Listeria monocytogenes*) bacteria. Eucalyptus oil displays also anti-inflammatory quality and anti-viral activity, e.g., against influenza A (H1N1) virus. Besides, this oil can potentially act as an Mpro inhibitor of COVID-19. What is more, it has been found to show phytotoxicity to *Sinapis arvensis* and *Raphanus sativus* [75,76,77,78,79,80,81]. Eucalyptol alone belongs to compounds having a potential for treatment of influenza [82]. Another compound, which is present plentifully in the aroma profile of two night-blooming species representing the genus Silene (*Caryophyllaceae*), namely *S. chlorantha* (40.5%) and *S. italica* (14.5%), is β-linalool. It is also present in *Osmanthus fragrans* var. thunbergi (27.71%) [83,84] and cardamom (0.44–11.0%) [85]. And this is mainly linalool that is responsible for the fragrance of the lychee fruit [86]. This compound, also one of the basic components of *Citrus reticulata* leaves essential oil, possesses promising attributes as an additive to anti-aging cosmetics for skin care [74]. Fumigation with linalool hinders significantly the growth of *Botrytis cinerea* mycelium and expansion of this pathogen on tomato fruit [87]. Thymol is the main component of essential oils emitted by plants falling within the family *Lamiaceae*. In *Thymus vulgaris* L. essential oil the percentage of this compound ranges from 10 to 64%. Numerous varied types of activity of thymol have been revealed, including anti-oxidant, anti-inflammatory, molocally anaesthetic, anti-nociceptive, scarring, anti-septic, anti-bacterial, anti-fungal, anti-cancerogenic, antispasmodic, anti-Leishmanial, anti-biofilm, anti-viral properties, and also its effect as a growth stimulator and immunomodulator. It also shows therapeutic attributes against different cardio-vascular, neurological, rheumatic and gastro-intestinal diseases [88,89,90,91]. What is more, thymol was found causing the weakening of *Nosema ceranae* individuals, infesting the honey bee *Apis mellifera*, and a decline in the productive and reproductive capabilities of their microsporidians [92]. As shown in our investigations and also other authors’ research Table S1 [22,28,29,30,31,32,33,34,35,36,37] addition of plant hormones to the breeding medium in plant in vitro cultures affects the composition and share of particular compounds in the case of various species of plants, *Hylocereus undatus* for example. Our results may be a starting point for further investigations and optimization to improve the potential of in vitro pitaya cultures for biotechnological production of valuable for numerous industries, such as pharmaceutical or plant protection ones, volatile compounds.

## 4. Materials and Methods

### 4.1. Biological Material

**Figure 1:**
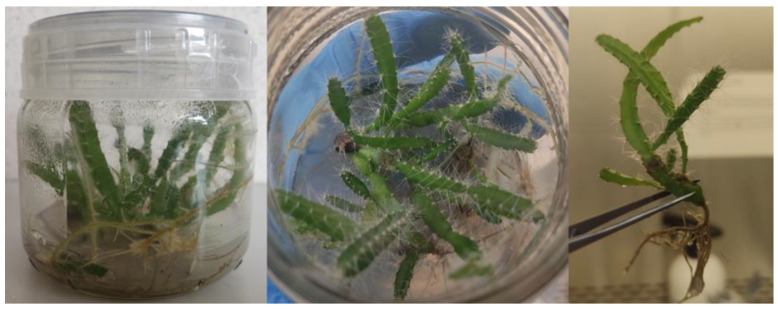
*Hylocereus undatus* motherplant in vitro cultures.

Initial material for chemical analysis were stems of *Hylocereus undatus* comprized within the plant collection run under in vitro conditions at the Faculty of Genetics, Plant Breeding and Seed Production (Figure 1). Two pools of explants bred on the MS (Murashige and Skoog) medium with an addition of different PGRs (BA—for multiplication and IAA—for elongation) with concentration 0.5 mg·L^−1^, which is the standard concentration used for preliminary in vitro studies. The multiplication of the material without a control (MS basic medium without hormones) was performed due to poor plant growth and vitality on it [7,93,94]. Then plants were selected for the analyses of volatile compounds.

### 4.2. Aroma Profiling

The analysis of the stem chemical composition was performed with the use of a gas chromatograph coupled with a mass spectrometer (single quadrupole mass spectrometer; gas chromatograph Shimadzu GC-MS QP 2020, Shimadzu, Kyoto, Japan) with using solid-phase micro extraction (SPME) technique. Each sample (~500 mg of fresh plant material respectively) was placed in a vial of the head-space type, volume of 20 mL. The polymer coating of the fibre was a mixture of divinylbenzene, WR carboxene and polydimethylsiloxane (DVB/C-WR/PDMS). The volatile extraction was performed at 80 °C for 10 min; before the extraction sample was preincubated for 10 min at extraction temperature. The desorption of extracted volatile was carried out in the apparathus injector for 3 min. The injector temperature was 250 °C. As the carrier gas, helium at flow 1.0 mL/min, with split 5, was applied. Separation was reached using a capillary column Zebron ZB-5 (30 m, 0.25 mm, 0.25 µm of stationary phase; Phenomenex, Torrance, CA, USA). The temperature program of the column was as follows: 50 °C, an increase by 4.0 °C min^−1^ to 130 °C, then 10 °C min^−1^ to 180 °C then 20 °C min^−1^ to 280 °C. The MS analysis was performed using scans from 40 to 300 *m*/*z* with the application of electron ionization (EI) at 70 eV. The analysis was carried out in four replications.

The compounds were identified with the help of two different analytic methods in order to compare the retention times of authentic chemical compounds (standard of saturated alkanes Supelco C_6_–C_30_) with the mass spectra acquired from the available library (Willey NIST 17, match indicator; 90%). The identification of compounds was performed through a comparison of the experimentally obtained linear retention indices calculated relative to the mixture of *n*-alkanes C_6_–C_30_ (SigmaAldrich, Saint Louis, MO, USA) and mass spectra with the ones available at libraries (NIST 17 Mass Spectral and Retention Index Library (NIST17) and NIST WebBook) or in literature [95].

### 4.3. Statistical Analysis

The results were expressed as the mean of the measurements and reported as mean ± SD (standard deviation). The data reported in the present study are the mean values of at least four replicates. A one-way analysis of variance (ANOVA) was conducted to verify the lack of significance of PGRs (plant growth regulators). The hypothesis assumed no impact of medium (supplemented with various PGRs) on the chemical content of pitaya. The significant differences were assessed at levels of 0.05 and 0.01. When an analysis of variance gave a significant result, Tukey’s honestly significant difference (HSD) test was performed to compare mean values [96]. Data obtained during aroma profiling were subjected to a one-way analysis of variance using Tukey’s test (*p* < 0.05). The obtained results were statistically analyzed with the use of the Statistica programme, version 13.3. Tukey’s HSD test was performed in those cases where the hypothesis was rejected.

## 5. Conclusions

The present study revealed effect of cytokinin BA (6-6-benzylaminopurine) and auxin IAA (indolyl-3-acetic acid) on modification of the volatile compounds profile in the overground part of the white pitaya plants coming from in vitro cultures. And thus, plant hormones display properties leading to alterations in the profile of volatile compounds. Furthermore, they possess a potential for increasing the percentage of particular valuable compounds in plants.

The hitherto conducted investigations were focused on analysis of compounds in particular parts of fruit of different species representing the genus *Hylocereus*, while only the present research pertains to volatile compounds contained in stems of the white pitaya. Our research, based on in vitro cultures, was performed in the context of future application of the technique itself to purposeful and directed modification of the chemical composition in plants.

## Figures and Tables

**Table 1:** Comparison of percentage use for volatile pitaya plants grown in vitro on two variants of MS medium with the addition of BA or IAA.

No.	Compound	Experimental Retention Index	Litrature Retention Index	BA [%]	SD	IAA [%]	SD
1	Hexanal	807	806	6.87 ^e^*	±1.13	4.53 ^ef^	±0.45
2	2-Hexenal	858	860	22.53 ^d^	±3.74	25.48 ^c^	±2.77
3	1-Hexanol	862	868	40.89 ^a^	±3.58	36.44 ^d^	±2.02
4	2-Heptenal, (*Z*)-	961	964	1.12 ^g^	±0.18	0.79 ^g^	±0.09
5	2-Hexenoic acid, methyl ester, (*E*)-	970	966	1.94 ^fg^	±0.84	0.99 ^g^	±0.49
6	1-Heptanol	974	970	-	-	0.24 ^g^	±0.25
7	1-Octen-3-ol	983	980	-	-	0.35 ^g^	±0.32
8	3-Octanone, 2-methyl-	987	985	0.59 ^g^	±0.44	2.67 ^fg^	±1.72
9	Hexanoic acid	989	981	0.91 ^g^	±0.69	1.05 ^g^	±0.53
10	Furan, 2-pentyl-	992	993	0.67 ^g^	±0.27	0.39 ^g^	±0.08
11	Octanal	1004	1007	-	-	1.04 ^g^	±1.01
12	*o*-Cymene	1029	1022	-	-	0.82 ^g^	±0.64
13	Limonene	1034	1031	1.26 ^g^	±0.31	0.95 ^g^	±0.22
14	Eucalyptol	1036	1033	1.66 ^g^	±0.11	0.93 ^g^	±0.18
15	Pyrazine, 3-methoxy-2.5-dimethyl-	1057	1054	0.39 ^g^	±0.26	0.43 ^g^	±0.09
16	1-Octanol	1074	1082	0.70 ^g^	±0.35	0.60 ^g^	±0.26
17	Heptanoic acid	1082	1078	0.45 ^g^	±0.30	-	-
18	Pyrazine, 2-methoxy-3-(1-methylethyl)-	1096	1093	1.27 ^g^	±0.09	2.07 ^fg^	±0.36
19	Linalool	1100	1103	1.49 ^g^	±0.24	0.57 ^g^	±0.07
20	Nonanal	1105	1104	0.67 ^g^	±0.17	1.30 ^g^	±1.29
21	Hexanoic acid, 2-ethyl-	1126	1123	-	-	0.85 ^g^	±0.64
22	Benzene, 1.2-dimethoxy-	1149	1149	1.13 ^g^	±0.23	0.78 ^g^	±0.12
23	Pyrazine, 2-methoxy-3-(1-methylpropyl)-	1175	1175	2.55 ^fg^	±0.98	2.45 ^fg^	±0.71
24	Octanoic Acid	1179	1182	0.66 ^g^	±0.35	0.98 ^g^	±0.40
25	Pyrazine, 2-methoxy-3-(2-methylpropyl)-	1182	1192	1.03 ^g^	±0.22	1.31 ^g^	±0.07
26	Ethanol, 2-(2-butoxyethoxy)-	1190	1196	-	-	0.36 ^g^	±0.23
27	Decanal	1205	1206	0.89 ^g^	±0.23	0.51 ^g^	±0.08
28	2-Decenal	1263	1270	-	-	0.60 ^g^	±0.76
29	Nonanoic acid	1277	1280	1.24 ^g^	±0.32	1.06 ^g^	±0.90
30	Thymol	1293	1291	0.67 ^g^	±0.11	1.60 ^g^	±1.18
31	Unknow 1	1359	-	0.74 ^g^	±0.37	0.65 ^g^	±0.20
32	Unknow 2	1375	-	0.55 ^g^	±0.22	1.49 ^g^	±0.69
33	Unknow 3	1379	-	1.07 ^g^	±0.61	0.63 ^g^	±0.59
34	Tetradecane	1399	1400	-	-	0.59 ^g^	±0.51
35	Geranyl acetone	1457	1452	-	-	0.27 ^g^	±0.05
36	1-Dodecanol	1478	1473	2.42 ^fg^	±1.12	1.33 ^g^	±0.26
37	β-Ionone	1490	1494	0.51 ^g^	±0.10	0.59 ^g^	±0.05
38	Octanoic acid, hexyl ester	1584	1580	0.53 ^g^	±0.28	-	-
39	Unknow 4	1638	-	0.43 ^g^	±0.44	0.78 ^g^	±0.09
40	Octyl ether	1667	1657	0.66 ^g^	±0.18	0.27 ^g^	±0.06
41	Unknow 5	1680	-	0.60 ^g^	±0.25	0.54 ^g^	±0.15
42	Norphytan	1707	1703	0.10 ^g^	±0.03	0.30 ^g^	±0.08
43	Phytan	1811	1811	0.84 ^g^	±0.58	0.41 ^g^	±0.16

* Values followed by the different letter are significantly different (*p* > 0.05, Tukey’s test); SD standard deviation.

## Data Availability

Data is contained within the article or supplementary material or data is available on request.

## Supplementary Materials

The following supporting information can be downloaded at: https://www.mdpi.com/article/10.3390/molecules28093843/s1, Table S1: Modification the chemical composition using chemical and physical factor in plants under the conditions.

## Sample Availability

Samples of the compounds are not available from the authors.
