# Supplementary material for: The Potential Use of Plant Growth Regulators for Modification of the Industrially Valuable Volatile Compounds Synthesis in Hylocreus undatus Stems
Source: Molecules. 2023 May 1;28(9):3843. doi: 10.3390/molecules28093843 (PMC10180215; doi:10.3390/molecules28093843)
Supplement: Supplementary file 1 [file molecules-28-03843-s001.zip › molecules-2346917-supplementary.pdf]

Table S1. Modification the chemical composition using chemical and physical factor in plants under the conditions

Species	Modifying factor/conditions	Effect	References
Hylocereus polyrhizus	Polyphenol oxidase, CYP76AD3 and 4,5-DOPA dioxygenase extradiol-like protein;<br><br>*in vivo*	Betaine biosynthesis	[29]
Hylocereus monacanthus	Autopolyploization;<br><br>*in vivo*	Reduction in the concentration of sugars and betacyanins, increase in the amount of amino acids, intermediates of the TCA cycle, organic acids and<br>flavonoids	[30]
Amaranthus caudatus	Cytokines<br><br>*in vivo*	Increase in the content of betacyanins	[42]<br><br>[43]
Amaranthus caudatus	Abscisic acid (ABA)<br><br>*in vivo*	Decrease in betacyanin content	[44]
Hylocereus undatus	Trypsin<br><br>*in vivo*	Fatty acid elongation<br>three unsaturated fatty acids were upregulated, while eight saturated fatty acids were downregulated	[31]
Hylocereus polyrhizus	Heat stress + 40°C<br><br>*in vivo*	Content increase glycerol tributanoate, cis-aconitate, L-isoleucine, and mesaconic acid	[32]
Hylocereus undatus	Induced wound<br><br>*in vivo*	Phenol biosynthesis	[33]
Solanum lycopersicum	Biotic and abiotic stress<br><br>*in vivo*	Increasing the accumulation of carotenoids	[45]
Hylocereus polyrhizus	Red light radiation of fruit<br><br>*in vivo*	Decrease in D-fructose, D-glucose, mannose, sorbose, D-turanose, glucopyranose and D-glucopyranoside<br><br>content of octadecanoic acid, hexadecanoic acid, eicosanoic	[34]

		acid, ethanedioic acid, pentanedioic acid and tyrosine<br><br>volatile compounds have changed significantly<br><br>content of hexanal, 2-hexenal, 2-heptenal and 4-heptenal significantly decreased, cyclohexenone, 1-hexanol significantly increased<br><br>2-hydroxy-cyclopentadecanone and 2-octenal acid increased significantly	
Hylocereus undatus	Red and blue light in a ratio of 1 : 2<br><br>*in vitro*	Accumulation of sucrose, glucose, fructose, fructose-6-phosphate, fatty acid and flavonoids increased significantly	[35]
Hylocereus polyrhizus	PEG-induced drought stress<br><br>*in vitro*	Increased osmolyte accumulation, lipid peroxidation and antioxidant enzyme activity	[36]
Hylocereus costaricensis	Elicitors (silver nitrate, yeast extract), amino acids, tyrosine, leucine<br><br>*in vitro*	Increasing the content of betaine	[37]
	Red light<br><br>*in vitro*	Enhanced betalain synthesis 3,8-fold and 4,8-fold	
Hylocereus polyrhizus	Tyrosine<br><br>*in vitro*	Supporting the accumulation of betacyanin, betaxanthin, phenol and flavonoids	[38]
Berberis vulgaris	Tyrosine<br><br>*in vitro*	Increase in betacyanin content	[46]
Hylocereus polyrhizus	Methyl jasmonate<br><br>*in vivo*	Fruit treatment increases betacyanin content and antioxidant activity	[39]
Hylocereus costaricensis	Low sucrose<br><br>*in vitro*	Stimulation of the activity of betalain compounds and antioxidants	[40]

Hylocereus polyrhzus	Salicylic acid *in vitro*	Increase in the production of betalains	[41]
Alternanthera tenella			[47]
Elaeagnus angustifolia		Increase in alpha-tocopherol synthesis	[48]
Vitis vinifera		Increasing anthocyanin synthesis	[49]
Andrographis paniculata		Increasing the synthesis of flavonoids	[50]
Mentha piperita L.	2-Isopentyladenine (2iP) and indolyl-3-acetic acid (IAA) *in vitro*	Increasing the content of mentofurolactone	[51]
Phoenix dactylifera L.	2-Isopentyladenine (2iP) and 2,4-dichlorophenoxyacetic acid (2,4-D) *in vitro*	Increasing the production of phenolic compounds, flavonoids	[52]
Vaccinium corymbosum L	Zeatin *in vitro*	Increasing the content of lipophilic compounds	[53]
Hordeum Spring barley	(6-(3-methoxybenzylamino)-9-(β-d-arabinofuranosyl)purine - cytokinin derivative *in vitro*	Grain yield increase	[54]
Artemisia argyi	Abscisic acid (ABA) *in vivo*	Stimulates the biosynthesis of many secondary metabolites, including phenylpropanoids, flavonoids, terpenoids, alkaloids	[55]
Calendula officinalis	Jasmonic acid *in vitro*	Increasing the synthesis of saponins	[56]
Solanum lycopersicum cv. Grape	methyl jasmonate *in vivo*	Induced the production of amino acids and fatty acids inducing the accumulation of α-tocopherol and β-sitosterol	[57]
Brassica napus L.	Gibberellic acid (GA3)	Increase in the content of oleic acid	[58]

	in vivo		
Nitraria tangutorum	Indolyl-3-acetic acid (IAA), abscisic acid (ABA) and gibberellic acid (GA3)<br><br>*in vivo*	significantly increased the content of osmotic regulatory substances (soluble sugar, soluble protein, and proline) and antioxidant enzymes (SOD and POD)	[59]
Nelumbo nucifera Gaertn	Abscisic acid (ABA)<br><br>*in vivo*	Increase in starch content	[60]
Lavendula angustifolia 'Luoshen'	Methyl jasmonate<br><br>*in vivo*	Increase in the content of volatile substances (monoterpenoids and sesquiterpenoids)	[61]
Citrus sinensis L. Osbeck	Methyl jasmonate<br><br>*in vivo*	Change in the content of volatile compounds increase in the emission of E-β-ocimene, indole	[62]
Quercus pyrenaica	Methyl jasmonate<br><br>*in vivo*	Overall composition did not differ but showed higher emissions of volatile compounds	[63]
